# Optimizing photoacoustic image reconstruction using cross-platform parallel computation

**DOI:** 10.1186/s42492-018-0002-5

**Published:** 2018-09-05

**Authors:** Tri Vu, Yuehang Wang, Jun Xia

**Affiliations:** Department of Biomedical Engineering, University at Buffalo, The State University of New York, Buffalo, USA

**Keywords:** Photoacoustic computed tomography, Graphics processing units, Parallel computation, Focal-line back-projection algorithm, MATLAB, Optical imaging

## Abstract

Three-dimensional (3D) image reconstruction involves the computations of an extensive amount of data that leads to tremendous processing time. Therefore, optimization is crucially needed to improve the performance and efficiency. With the widespread use of graphics processing units (GPU), parallel computing is transforming this arduous reconstruction process for numerous imaging modalities, and photoacoustic computed tomography (PACT) is not an exception. Existing works have investigated GPU-based optimization on photoacoustic microscopy (PAM) and PACT reconstruction using compute unified device architecture (CUDA) on either C++ or MATLAB only. However, our study is the first that uses cross-platform GPU computation. It maintains the simplicity of MATLAB, while improves the speed through CUDA/C++ − based MATLAB converted functions called MEXCUDA. Compared to a purely MATLAB with GPU approach, our cross-platform method improves the speed five times. Because MATLAB is widely used in PAM and PACT, this study will open up new avenues for photoacoustic image reconstruction and relevant real-time imaging applications.

## Background

Photoacoustic imaging is an emerging modality, which is well-known for overcoming the light diffusion limit by converging light absorption into sound [1]. Upon irradiation by laser or radiofrequency pulses, tissue will experience thermos-elastic expansion, which generates acoustic waves to be detected by transducers. Capitalizing on non-ionizing light illumination and rich optical contrasts, photoacoustic imaging possesses advantages in term of safety, penetration depth, and tissue contrast [2]. Photoacoustic computed tomography (PACT), in particular, employs higher energy pulses and wide-field scanning and is capable of capturing 3D structures in a wide range of scales, from vasculatures to organs [3, 4]. This character gives PACT an outstanding advantage over other tomography modalities [5].

Despite its immense possibility [6–8], PACT is limited by the extensive 3D computation. For example, to reconstruct a 200 × 430 × 200 matrix, it takes half an hour on GPU-based MATLAB on our PC with an NVIDIA Titan X, using the focal-line-based 3D reconstruction algorithm [9]. Even with the fact that MATLAB is not time-efficient, this processing time is still considerable, placing a burden on the “pipeline” of 3D PA studies. Since reconstruction is the very “front door” component in this “pipeline”, long reconstruction time leads to delay in the overall research process.

Current efforts on shortening PA reconstruction range from algorithm development to hardware improvement. In terms of algorithm development, fast Fourier transform-based (FFT) reconstruction [10] has succeeded at improving reconstruction speed. On the other hand, in terms of hardware enhancement, with the recent boom in graphics processing units (GPU), parallel computation has been widely used in various medical tomography modalities, such as PA [11–14], CT and MRI [15–18]. Because PA reconstruction involves mostly linear computation which is straightforward for being parallelized, GPU becomes a suitable solution for improving the computation time [11–14]. Kang et al. [11] combined both FFT reconstruction with GPU to show a significant improvement of 60 times compared to single-thread CPU on optical-resolution photoacoustic microscopy (OR-PAM) with 500 × 500 pixels. Impressive improve in performance also proved in 3D reconstruction. For instance, Wang et al. implemented GPU-based image reconstruction on C and demonstrated an improvement of 1000 times in comparison with CPU [19]. Luis et al. even managed to perform 4D PA imaging with 120 × 120 × 100 voxels and achieved a speed of 51 frames per second [20]. However, all these studies focused on the reconstruction efficiency and neglected the front-end simplicity of user interaction, which is also important in PA studies.

To improve the user-friendliness of image reconstruction, here we propose a cross-platform image reconstruction approach. Our solution is different from previous studies in a sense that it spans across two programming platforms – MATLAB and C++ on CUDA API. This MATLAB/C++/CUDA code (MCCC) combines the simplicity of MATLAB and the time-efficiency of C++. It can tremendously assist PA research because most of current PA systems heavily depend on MATLAB. In details, the reconstruction code is back-projection-based, with pre- and post-processing steps performed in MATLAB and reconstruction loops executed in CUDA/C++, through MEXCUDA functions. Validating images are then reconstructed using the MATLAB/CUDA-without-C++ code (MCC), MATLAB-without-GPU code (MWGC), and our MCCC in this study. MCC does not perform any computation in C++, and MWGC processes all the steps on CPU only. They are used to compare with MCCC to see if our cross-platform method reduces the reconstruction time. Successfully, our solution is able to shorten this processing time to one-fifth while keeping the same image quality comparing to the MCC.

## Methods

### Reconstruction method – focal-line-based back-projection algorithm

In PACT, the universal back-projection (UBP) is frequently used for 3D image reconstruction [21]. Details of this reconstruction method are described by the following formula:

$$ {p}_0\left(\overrightarrow{r}\right)=\frac{1}{\Omega_0}\underset{S}{\int }d\Omega {\left.\left[2p\left(\overrightarrow{r_d},t\right)-2t\frac{\partial p\left(\overrightarrow{r_d},t\right)}{\partial t}\right]\right|}_{t=\left|\overrightarrow{r_d}-\overrightarrow{r}\right|/{v}_s} $$

Here, ${p}_0\left(\overrightarrow{r}\right)$ is the initial PA pressure at $\overrightarrow{r}$, $p\left(\overrightarrow{r_d},t\right)$ is the acoustic pressure at $\overrightarrow{r_d}$, and delay time *t* is calculated from the travel time $\left|\overrightarrow{r_d}-\overrightarrow{r}\ \right|/{v}_s$, in which *v*_*s*_ is the speed of sound in tissue (1.54 m/msec). *Ω*_0_ is the solid angle spanning over the transducer surface *S*. The universal back-projection algorithm is developed based on point-like transducers and is inaccurate for focused transducers, such as linear transducer arrays with a focus along the axial direction. In this case, because of the element aperture, time delay cannot be computed directly from the point source to the center of the element. The focal-line reconstruction algorithm addresses this issue by utilizing a focal line which goes through the foci of all transducer elements. The travel path (time of arrival) of any point in 3D space is quantified based on its intersection with the focal line: only the path that goes across the focal line gives the strongest response in the transducer. Detailed descriptions of this method can be found in [9, 22].

### MEXCUDA function generation

As aforementioned, MATLAB is used as the main platform for pre- and post-processing the data and all the extensive computation process is performed in C++. Such that, we need to establish a “gateway” between CUDA/C++ and MATLAB. MEXCUDA function offers a perfect solution for this connection. It is a convenient way to take input from MATLAB to C++, perform calculation in C++, and then take the output back to MATLAB. In details, MEXCUDA is the expansion of MATLAB mex function that utilizes C/C++ for execution using C++ MEX API. The difference between mex and MEXCUDA is that MEXCUDA is compiled by the NVIDIA CUDA compiler (nvcc), enabling GPU execution on C++ for improved performance.

We first need to generate a MEXCUDA function before calling it in MATLAB. The source code for the MEXCUDA function is a CU file which is written in C++ for CUDA. The CU file has the following main building blocks. The first block is initialization with two purposes. First, it prepares the code with MathWorks’ GPU library by calling mxInitGPU from the mxGPU API. Secondly, it creates mxGPUArray objects (mxGPUArray is a CUDA class to contain GPU arrays) to store gpuArray inputs from MATLAB and an output matrix “*pa_img*” representing the reconstructed image. The next block of code is parallel computation. It contains several kernel functions on the device code to calculate *pa_img* from the input mxGPUArray objects in parallel. The last block of the CU code is finalization. It includes functions to deliver *pa_img* back to MATLAB code and to destroy the GPU matrices to save memory. From this source code, we create the compiled MEXCUDA function by using the mexcuda command in MATLAB. This final MEXCUDA function is in mexw64 type, which is a nvcc-compiled code for the 64-bit Windows operating system. This function can be called directly in Matlab as a subfunction.

**Fig. 1:**
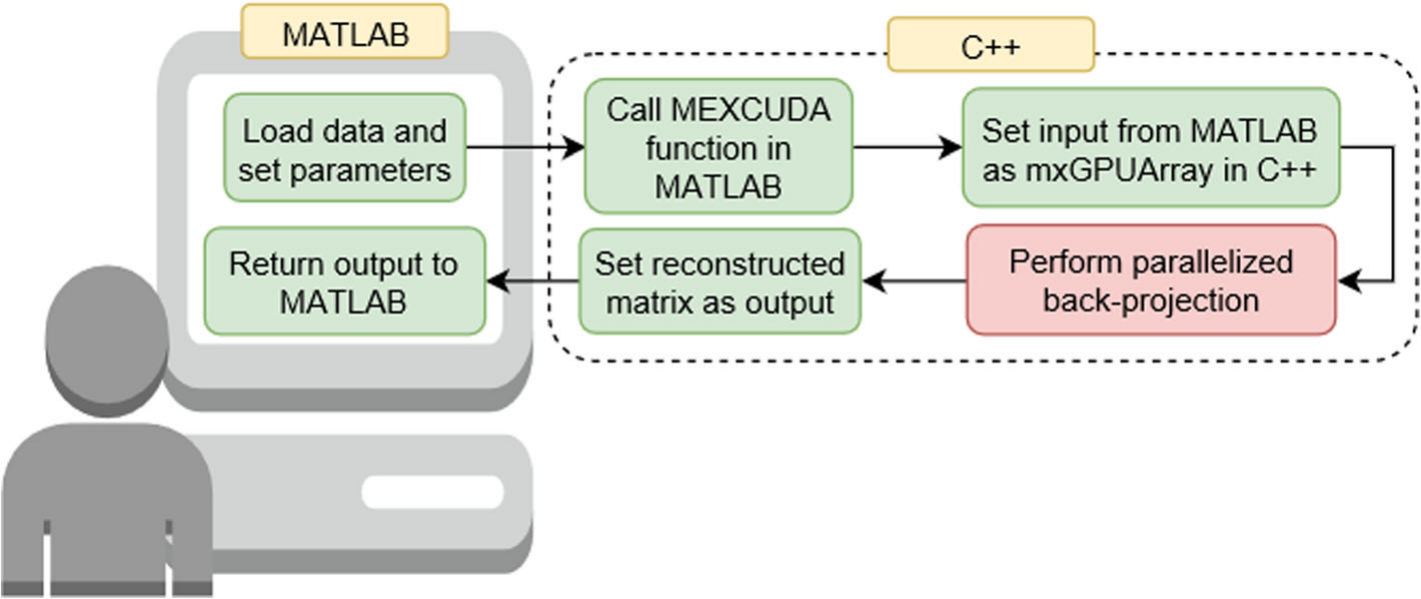
Overall flowchart of a MEXCUDA function

The workflow of a function execution by MEXCUDA is demonstrated in Fig. 1. First, in the MATLAB front-end code, users load raw data, convert CPU-based matrices into GPU matrices, and set reconstruction parameters. Then, users send inputs to MEXCUDA function. After executing through the building blocks mentioned above, this function returns the output as the final reconstructed image to MATLAB. Finally, with post-processing steps in MATLAB, users are able to visualize and examine the reconstructed 3D structure.

### Heterogeneous computing in CUDA/C++

**Fig. 2:**
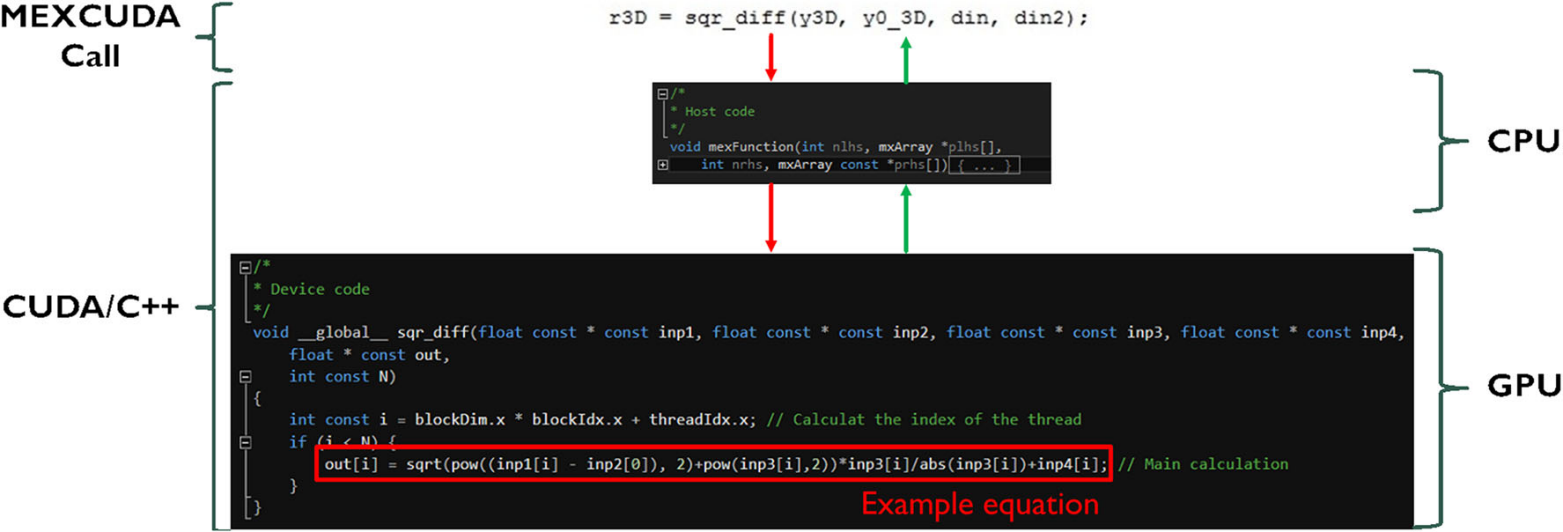
Example process flow of the heterogeneous computing. In this figure, we create a sample MEXCUDA function for calculating the radius matrix of a scanned region to each transducer elements. The host code (CPU) is in charge of initialization and finalization blocks such as reading the input from MATLAB, performing condition checks, and allocating memory for input and output data. Device code in GPU is responsible for computing the radius matrix in parallel from required inputs (parallel computation block)

The process flow executed in C++ employs a widely-known programming method called heterogeneous computing in order to maximize the performance. GPU, despite having excellent computing ability by calculating each matrix value in parallel, cannot perform both traditional serial and CPU-based tasks effectively, such as checking input compatibility, pre-allocating memory, and creating output arrays. On the other hand, CPU is faster at handling these steps so it is better suited for pre- and post-processing data. Such that, CPU is employed in the initialization and finalization blocks, while GPU is exploited in the parallel computation block. This processing flow is presented in Fig. 2.

### Validating experiments

**Fig. 3:**
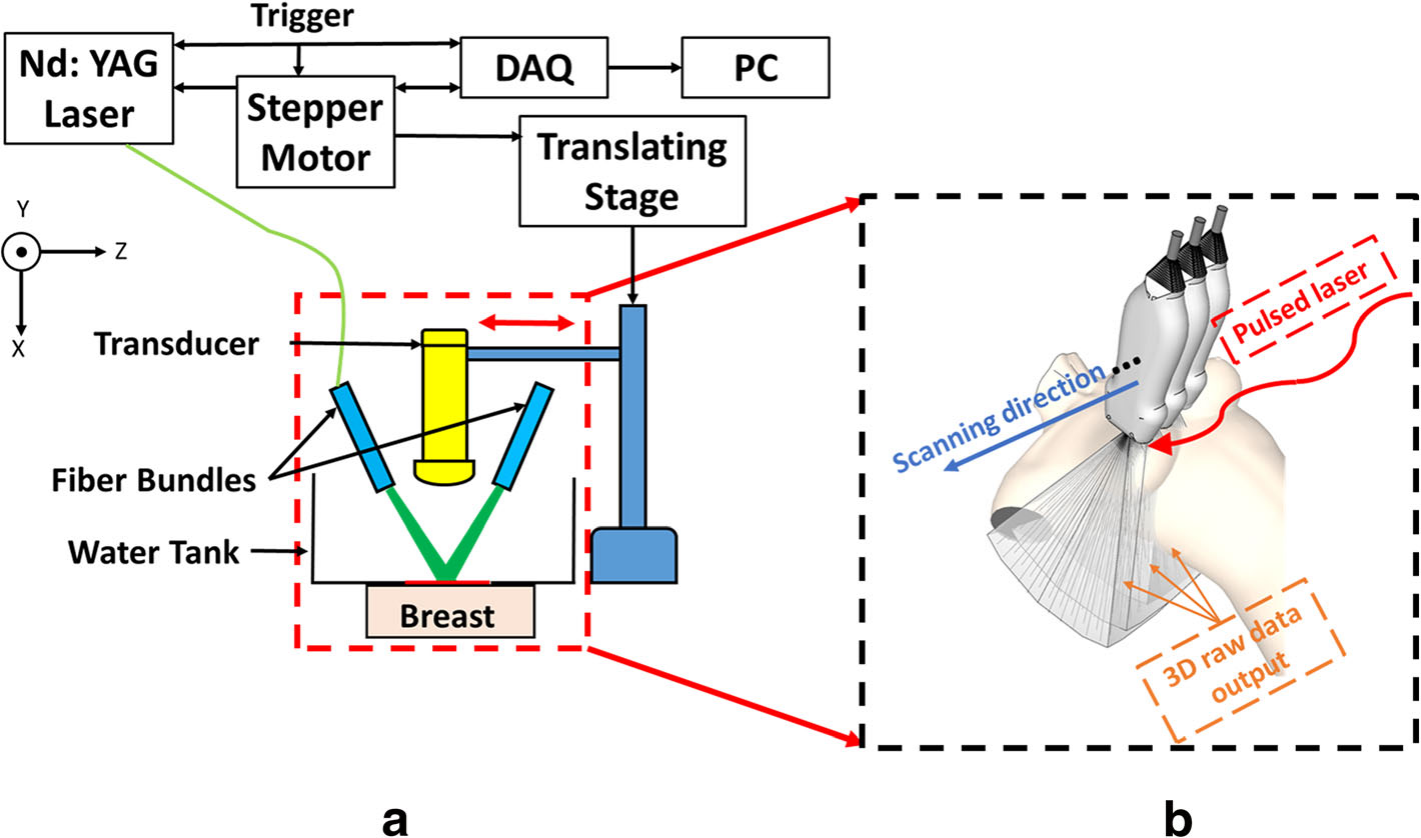
Schematic drawing of PACT setup. **a** A 2D schematic showing all major components. **b** A 3D illustration of the scanning process. The laser beam causes the tissue to expand and release acoustic waves which are captured by the transducer. This process happens continuously along the moving (scanning) direction

To evaluate the efficiency of the optimized code, we scanned a breast of a healthy volunteer to acquire 3D vascular data. The human imaging study was performed in compliance with the University at Buffalo IRB protocols. The PACT imaging system contains three main parts: a 10-ns-pulsed Nd:YAG laser with 10 Hz pulse repetition rate and 1064 nm output wavelength, a customized linear array with 128 elements and 2.25 MHz central frequency, and a Verasonics’ Vantage data acquisition system with 128 receive channels. The light illumination was achieved through a bifurcated fiber bundle with 1.1-cm-diameter circular input and two 7.5-cm-length line outputs (Light CAM #2, Schott Fostec). During the experiment, the input laser energy was around 800 mJ/pulse and the efficiency of the fiber bundle is 60%, so that the laser output from the fiber bundle is around 480 mJ/pulse. Since the size of the laser beam on the object’s surface was approximately 2.5 cm × 8.0 cm, the laser intensity is 30 mJ/cm^2^, which is much lower than the safety limit of 100 mJ/cm^2^ [23]. The transducer was scanned along the elevation direction over 40 mm at 0.1 mm step size. The entire imaging area is 8.6 cm (lateral width of the probe) × 4 cm (scanning distance). A schematic of the experimental setup is illustrated in Fig. 3. Following data collection, we performed 3D focal-line reconstruction with MCCC, MCC and MWGC for comparison.

## Results

We reconstruct the image using MCC, MCCC and MWGC methods for comparison of processing time and image quality. All the reconstructions are carried out in our PC with an NVIDIA Titan X GPU (Pascal architecture) and Intel Core i5-6400 CPU.

**Fig. 4:**
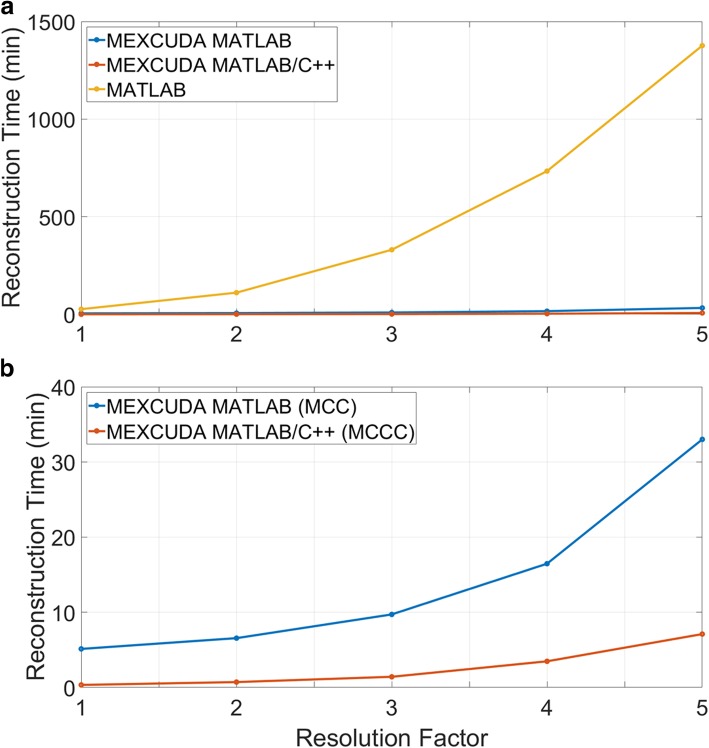
**a** Comparison of reconstruction time between MCC, MCCC and MWGC and **b** a close look into reconstruction time difference between MCC and MCCC codes with different RF

In terms of reconstruction time, even though it is already supported by GPU for parallel programming, MCC reconstruction still shows a costly computing time. It takes more than 30 min with a resolution factor (RF) of five for a volume of 200 × 430 × 200 voxels. Here, RF is the reciprocal of the voxel size (in mm). Reducing this number can reduce the reconstruction time to 400 s as shown in Fig. 4 with the loss of resolution as a tradeoff.

**Fig. 5:**
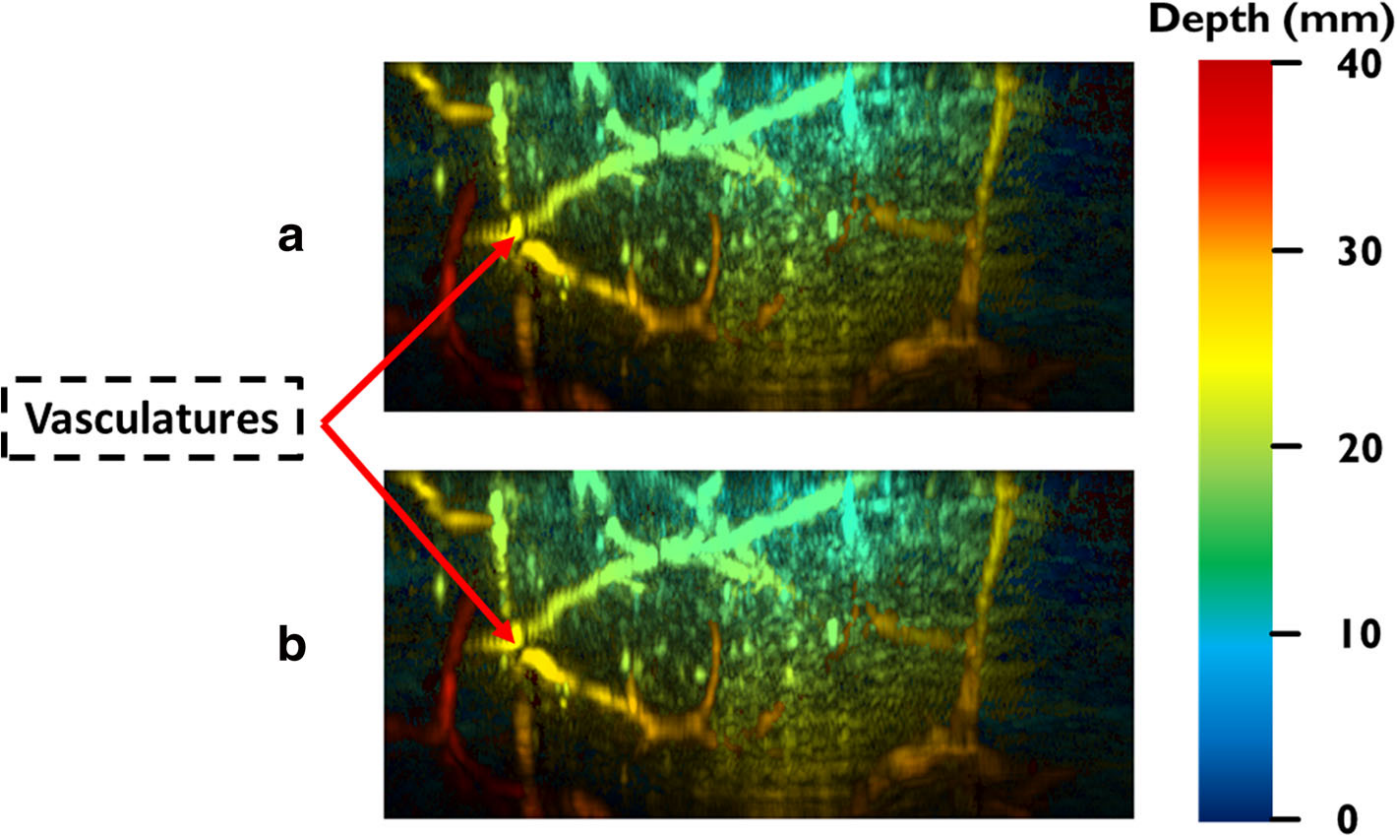
Comparison of depth-encoded photoacoustic images reconstructed by the (**a**) MCC and (**b**) MCCC

The results clearly show that C++ has played a vital role to shorten the reconstruction time. Overall, for RF of 5, computing time in MCCC is reduced by almost five-fold in comparison with MCC, from 33 to 7 min. Expectably, both of the codes with GPU support (MCC and MCCC) outperform the reconstruction without GPU (MWGC) which takes up to 1376 min as shown in Fig. 4(a). At RF of 2, MCCC has ten times shorter processing time than the duration of MCC as shown in Fig. 4(b). In terms of image quality, because the reconstruction methods are the same, there are no changes from those images created by MCC and MCCC as indicated by Fig. 5. This fact proves that there is no tradeoff between reconstruction accuracy and processing time. From the perspective of the users, as aforementioned, there is no need for modification of parameters or calculations in the source code in C++ because it is used as a predefined sub-function. With this fact, the simplicity nature of the front-end MATLAB code is maintained.

## Discussion and conclusion

To summarize, in this paper, we propose a novel way to optimize 3D reconstruction for PACT using cross-platform MATLAB/C++ code on CUDA. Our approach, utilizing C++/GPU reconstruction function, manages to significantly reduce the reconstruction time by five times compared with the performance of the MATLAB/GPU code. On the other hand, it maintains the simplicity of user interaction in MATLAB front-end side. Our method paves the way for future 3D reconstruction optimization for PACT on cross-platform MATLAB/C++ that benefits further PACT research which depends heavily on MATLAB.

Future work for this project will focus on further decreasing the reconstruction time by cutting down the number of iterations in the source code. The current reconstruction is still processed through a significant amount of loops based on the number of transducer elements and scanning lines. Instead of going through 128 (number of elements) × 400 (number of lines) loops, we should find a solution to perform calculation all at once if possible. For example, all the input data for each loop can be allocated to all available GPU memory and be executed in parallel. However, the limitation of this approach is that a huge amount of memory will need to be deployed, making it only viable for small 3D PA structures. Other than that, reducing the number of iterations can be achieved by having only either 128 loops based on transducer elements or 400 loops based on lines.
